# Enriched rehabilitation on brain functional connectivity in patients with post-stroke cognitive impairment

**DOI:** 10.3389/fneur.2024.1503737

**Published:** 2025-01-07

**Authors:** Yaping Huai, Weiwei Yang, Yichen Lv, Kui Wang, Hongyu Zhou, Yiqing Lu, Xiaoyun Zhang, Yaze Wang, Jibing Wang, Xin Wang

**Affiliations:** ^1^Department of Rehabilitation Medicine, Shenzhen Longhua District Central Hospital, Shenzhen, Guangdong, China; ^2^Department of Rehabilitation Medicine, Northern Jiangsu People's Hospital, Yangzhou, China

**Keywords:** enriched rehabilitation, post-stroke cognitive impairment, dorsolateral prefrontal cortex, functional connectivity, dominant hemisphere

## Abstract

**Objective:**

This study aims to observe the effect of enrichment rehabilitation (ER) on cognitive function in post-stroke patients and to clarify its underlying mechanism.

**Methods:**

Forty patients with post-stroke cognitive impairment (PSCI) meeting the inclusion criteria were randomly assigned to two groups: conventional medical rehabilitation (CM group) and ER intervention (ER group). All patients underwent assessments of overall cognitive function, attention function, and executive function within 24 h before the start of training and within 24 h after the 8 weeks of training. We investigated the altered resting-state functional connectivity (RSFC) with the right dorsolateral prefrontal cortex (DLPFC) in patients with PSCI following ER training through functional magnetic resonance imaging (fMRI). Additionally, twenty people undergoing routine physical examinations in the outpatient department of our hospital were selected as the healthy control (HC) group.

**Results:**

Before training, both groups of PSCI patients exhibited significant impairment in overall cognitive function, attention function, and executive function compared to the HC group. However, there was no significant difference between the two PSCI patient groups. Following 8 weeks of treatment, both PSCI patient groups demonstrated substantial improvement in overall cognitive function, attention function, and executive function. Moreover, the ER group exhibited greater improvement after training compared to the CM group. Despite the improvements, the cognitive behavioral performance assessment scores of both PSCI patient groups remained lower than those of the HC group. RSFC analysis in the ER group revealed strengthened positive functional connectivity between the right DLPFC and the left superior frontal gyrus (SFG) and left anterior cingulate gyrus (ACG), along with decreased functional connectivity between the right DLPFC and the right superior temporal gyrus (STG) and right precentral gyrus post-ER intervention.

**Conclusion:**

ER intervention is more effective than conventional medical rehabilitation in improving the cognitive function of PSCI patients, potentially by augmenting the FC between the right DLPFC and dominant cognitive brain regions, such as the left SFG and left ACG while attenuating the FC between the right DLPFC and non-dominant hemisphere areas including the STG and precentral gyrus within the right hemisphere.

## 1 Introduction

Post-stroke cognitive impairment (PSCI) is one of the most common sequelae of stroke (1). The cognitive challenges experienced by patients often manifest in various sub-domains such as attention, executive function, and memory, among others, contributing to difficulties in perceiving and adapting to the external environment (2, 3). These impairments not only hinder the patient’s comprehension of the rehabilitation therapist’s language but also impede the accurate execution of given instructions, significantly diminishing the efficacy of rehabilitation for limb dysfunction, swallowing issues, and other related impairments (3). Further, these cognitive deficits can lead to a decline in self-care and work capabilities, social function impairment, and mental health issues (4, 5). Studies have demonstrated a strong correlation between PSCI and an elevated risk of stroke recurrence, along with a heightened prevalence of post-stroke depression (6, 7). In addition, individuals with PSCI pose an added burden on their family members. Caregivers of PSCI patients are reported to be more susceptible to symptoms of depression or anxiety compared to those caring for stroke patients without cognitive decline. Within five years of the stroke incident, 20% of caregivers experience symptoms of anxiety, and 25% develop symptoms of depression, intensifying the burden on both the family and society at large (8, 9). Therefore, tackling the urgent requirement for effective rehabilitation training for PSCI remains a significant challenge in clinical practice.

Enrichment rehabilitation (ER) intervention represents a comprehensive rehabilitation approach that integrates environmental enrichment with task-oriented exercises (10). ER training could increase the level of sensory stimulation, cognitive activity, and organic motor function by placing subjects in more complex existential and social interaction scenarios, utilizing the novelty and complexity of the environment and multiple activities (11). This training strategy aims to enhance positive feedback and input to the central nervous system by creating a diverse and varied environment. It employs task-oriented training methods encompassing motor and sensory stimulation, cognitive activities, and social interactions. Studies have shown that ER interventions can enhance the secretion of nerve growth factor, brain-derived neurotrophic factor, nerve regeneration-related protein, etc., resulting in a long-term potentiation effect, enhancing the proliferation of neural stem cells, increasing the number of dendritic spines, realizing the reorganization of neuronal structure and function, and promoting the recovery of motor, sensory and cognitive functions (12–15). Some studies have proved that the theoretical basis of ER training is to foster the change of functional brain areas, enhance neuroplasticity, and ameliorate impaired functions (10, 16). Several recent studies have further demonstrated the rehabilitative potential of ER in improving both motor and cognitive dysfunction associated with various central nervous system conditions, including stroke, Parkinson’s disease, and others (17–19).

The resting-state network (RSN) is acknowledged as a structured system promoting the transmission of brain information, facilitating efficient information processing within and between relevant functional regions of the brain (20, 21). The statistical dependence between brain functional resting-state networks can be quantified using functional connectivity (FC), which also allows for the assessment of the organizational pattern and alterations in specific connections within these networks in the context of disease (21). Network-based statistical analysis revealed that post-stroke cognitive impairment was linked to whole-brain network dysfunction, involving 167 regions and 178 connections (22). This dysfunction resulted in a functional disconnection of brain regions associated with cognitive function, such as the frontal lobe and temporal lobe (22).

However, there is limited literature on the impact of ER intervention on brain FC in patients with PSCI. This study aims to examine the influence of ER on cognitive function and brain FC in PSCI patients and to provide clinical experimental evidence supporting the application of ER in cognitive rehabilitation.

## 2 Materials and methods

### 2.1 Ethical approval

The study protocol was approved by the Ethics Committee of Northern Jiangsu People’s Hospital Affiliated to Yangzhou University (approval no. 2018021). All participants provided written informed consent before study enrollment.

### 2.2 Participants recruitment

Forty patients diagnosed with PSCI and treated at the Northern Jiangsu People’s Hospital Affiliated to Yangzhou University from January 2020 to December 2021 were randomly allocated into two groups: a conventional medical treatment (CM) group (*n* = 20) and an ER group (*n* = 20). Utilizing the digital random method, 20 healthy subjects undergoing routine physical examinations in the outpatient department of the same hospital during the corresponding period were chosen to constitute the healthy control (HC) group. The general information of the three groups is presented in Table 1. The selection of sixty subjects adhered to specific inclusion and exclusion criteria:

**Table 1:** Demographics and main baseline characteristics of subjects categorized by study group.

Group	Number of cases	Age (years)	Gender	
			Male (*n*)	Female (*n*)
HC	20	51.67 ± 3.14	12	8
CM	20	50.98 ± 3.28	12	8
ER	20	51.02 ± 3.35	12	8

HC, Healthy Control; CM, Conventional; ER, Enriched Rehabilitation; N, Number.

Inclusion criteria for PSCI patients: All patients with cerebral infarction met the diagnostic criteria of cognitive impairment following cerebral infarction as outlined in the diagnostic criteria for cerebrovascular disease (23) and were assessed for enrollment in the study based on the following inclusion criteria (24): patients with right-handedness who experienced their first-ever ischemic cerebrovascular stroke (lesion in the left internal carotid artery system confirmed by MRI); initiation of rehabilitation treatment 1 to 2 months after the onset of stroke, with a Chinese version of the Montreal Cognitive Assessment (MoCA) score ranging from 20 to 23 within 24 h before treatment; ability of the patient to at least complete the basic activities and communication of ER with assistance; aged between 46 and 55 years with a minimum of 12 years of education.

Exclusion criteria for PSCI patients: Patients with a history of multiple strokes or other psychiatric and neurological conditions, including but not limited to brain trauma, Parkinson’s disease, mental disorders, hearing impairment, visual impairment, and severe cognitive impairment, were excluded. Additionally, individuals with a history of alcohol and substance abuse, poor adherence, and those unable to move autonomously, along with other medical conditions that could impede the effective implementation of ER intervention, were also excluded.

Inclusion criteria for healthy subjects: Twenty healthy subjects fulfilled the following inclusion criteria: participants must be conscious and have no history of mental illness or diseases of the nervous system; aged between 46 and 55 years with a minimum of 12 years of education. MoCA score within 24 h of the physical examination ranging from 28 to 30 points.

Exclusion criteria for healthy subjects: All healthy participants were required to be free from any neurological disorders as determined by a thorough physical examination. Additionally, they were expected to have no serious heart and kidney diseases, malignant tumors, or other conditions affecting cognitive and emotional functioning.

### 2.3 Basic demographic information

As shown in Table 1, no statistically significant differences were observed among the three groups concerning age and gender (P>0.05). Each group consisted of 12 males and 8 females. The age distribution was as follows: the average age of the HC group was 51.67 ± 3.14 years, the CM group was 50.98 ± 3.28 years, and the ER group was 51.02 ± 3.35 years.

### 2.4 Study schedule

The treatment protocol for all PSCI patients involved the administration of conventional medications, including antihypertensive agents, lipid-lowering drugs, and cognitive enhancers. In addition to these medications, the patients in CM group received one hour of routine rehabilitation training in the morning and afternoon each day, whereas the ER group received one hour of enriched rehabilitation training in the morning and afternoon each day. Both groups received rehabilitation training 6 days a week for 8 weeks.

The content of conventional cognitive rehabilitation training primarily encompasses attention, memory, logical thinking, calculation ability, executive function, and other cognitive sub-domains (24). The training comprised individualized single or multi-module integration tailored to patients following a cognitive assessment by the therapist. For instance, attention training involved promptly identifying two similar images or texts with differences. In auxiliary exercises, patients were guided to organize their daily activity plan execution or the sequence of specific tasks. Additionally, logical thinking training included tasks such as sequencing pictures in the correct story order and more.

In the ER group, therapists were required to create a diverse and dynamic environment, utilizing multi-sensory stimulation, cognitive activities, social engagements, and task-oriented training for comprehensive rehabilitation. The approach included the following components:

Enriched Environmental Stimulation: Utilizing multimedia equipment such as computers with internet connection and virtual reality technology, as well as other relevant equipment to design a variety of visual, auditory, olfactory, tactile and other multi-sensory stimulation projects. This allowed patients to experience various sensory stimuli triggered by differences in colors, smells, hardness, and more. For instance: ①Olfactory stimulation: Participants engaged in a 5-min odor identification task, involving the sniffing of two bottles of perfume with distinct scents, aiming to accurately name them. ②Auditory stimulation: Virtual reality equipment generated a variety of sounds, including animal calls and traffic horns. Participants were instructed to participate in blind listening, closing their eyes to accurately identify the source or describe the characteristics of each sound.

Cognitive Function Training: With the assistance of therapists, patients engaged in cognitive-related activities indoors using personal devices. Activities included reading books, listening to or humming music, browsing web pages of interest, playing regular card games, et al.

Task-oriented Exercise Training: ①Specific occupational therapy tasks were employed to strengthen the patient’s cognitive training for daily living. For example, patients were tasked with using the affected hand to select a specified color of water glass from three different colored glasses or filling a specific pattern with a designated color brush. ②To make physiotherapy more engaging and enhance functional motor training of the lower extremity, competitive elements were introduced among multiple patients during hip bridge exercises. Additionally, incorporating specific power bicycle training intensities based on different music rhythms can also be beneficial.

Social Activities: ①Patients and their family members went to the supermarket together. Once inside, patients independently selected and purchased the prescribed brand and quantity of goods, and settled the bill. ②Ensuring their safety, patients designed a route, with family members guiding them to take a bus to a designated city location, and then returning the same way. ③Participation in multiplayer games such as cards, board games, or table tennis, as well as engaging in group discussions with other patients or family members. ④Involvement in any other activities that patients enjoy doing with friends or family, such as watching movies or dancing, et al. In these activities, patients had the flexibility to choose and alternate between 2–3 items each week.

All patients underwent cognitive function tests within 24 h before the start of formal treatment and within 24 h after the completion of all treatment sessions. In addition, patients in the ER group underwent rs-fMRI examination both before and after the training period. The HC group completed the cognitive function test within 24 h after the physical examination.

### 2.5 MRI data preprocessing and analysis

Structural MRI and rs-fMRI were performed in the ER group before and after the intervention using a GE3.0 T MRI scanner. During scanning, all subjects were instructed to remain awake, keep their eyes closed, maintain a fixed head position, and rest without focusing on any specific thoughts. High-resolution structural images were acquired by employing a magnetization-prepared rapid gradient echo (MP-RAGE) sequence (25, 26): TR/TE/TI 1900/3.39/1100 ms, 7° flip angle, 240 mm × 240 mm mm field of view (FOV), 256 × 256 matrix, 176 axial slices, 0.9375 mm thickness, with an acquisition time lasting 4 min. The rs-fMRI scans were obtained using a gradient echo-echo-plane imaging sequence: 31 axial slices, 4 mm thickness, TR/TE 2000/30 ms, 90° flip angle, 240 mm (2) FOV, 64 × 64 matrix, with an acquisition time lasting 8 min.

Statistical Parametric Mapping^1^ and the RESTPLUS software^2^ were employed for preprocessing the resting-state functional MRI (rs-fMRI) data. Initially, we discarded the first 15 images for each subject to guarantee data stability. Subsequently, we corrected slice timing for the remaining images. Following this, we corrected for motion by aligning each volume to the average of all volumes. After assessing head motion parameters, we ensured that head displacement did not surpass 3 mm and the rotation angle did not exceed 3° for any patient. Then, we utilized Advanced Normalization Tools software for spatial normalization. Initially, we registered each subject’s T1 structural images to their mean functional image and mapped corresponding lesions onto it. Next, we registered the T1 structural image to the Montreal Neurological Institute and Hospital (MNI) standard space. Afterward, we applied the nonlinear transformation parameters acquired from the previous step to each motion-corrected volume, yielding each subject’s functional image in MNI standard space, and then resampled the spatially normalized functional image to a voxel size of 3 mm × 3 mm × 3 mm. Ultimately, we applied Gaussian smoothing with a full width at half maximum of 6 mm to all voxels. Additional denoising steps were carried out, including detrending and regressing out noise covariates such as motion-related parameters (i.e., Friston-24 model), white matter signals, cerebrospinal fluid signals, and other confounding variables. The data was filtered within a frequency range of 0.01 to 0.08 Hz.

## 3 Outcome measurement

### 3.1 Cognitive function measurement

The MoCA was used to assess the overall cognitive function, which involves the evaluation of attention, memory, execution, calculation, and other cognitive functions (24). The maximum score of the test was 30 points, with a score of ≥26 points considered normal in the general population.

The Trail Making Test (TMT) was used to evaluate executive function (27). In this study, the time required to complete TMT-A and TMT-B served as the evaluation indices. Attention function was estimated through the Symbol Digit Modalities Test (SDMT) (28), where the final score was determined by the number of correct modalities filled in within 90 s, excluding those filled in during practice.

## 4 Statistical analysis

The SPSS 22.0 statistical software package was used for data sorting and analysis. Quantitative data with a normal distribution were represented as mean ± standard deviation ( $\bar{x}+s$ ), while measurement data with a skewed distribution were represented using the median and interquartile range. Qualitative data (gender composition ratio) were compared between groups using a corrected chi-square test. Differences in age and cognitive function levels among the three groups were compared using one-way ANOVA. Paired *t*-test was used to compare the cognitive function of PSCI patients before and after treatment. *p* values less than 0.05 were considered to denote significant statistical differences (significance level: *α* = 0.05).

Statistical Parametric Mapping (SPM8) was utilized to process the rs-fMRI data. Participants with greater than 3.0 mm of translation or 3.0 degree of rotation in any direction were excluded. Following the normalization of anatomical images using Montreal Neurological Institute (MNI) templates, FC analysis was conducted using the Resting-State fMRI Data Analysis Toolkit (REST) (29).

The right DLPFC (coordinates: x = 45, y = 36, z = 21, with the mean signal of each voxel within the 6 mm radius sphere computed) was selected as the seed point and the region of interest (ROI). A voxel-wise FC analysis of each voxel was then performed for the fMRI data. The FC of each subject between each seed region and ROI was calculated and converted into Z-maps. Inter-group analysis involved individual single-sample *t*-tests for each group, followed by merging the results of each group into a mask. Subsequently, a two-sample *t*-test was performed between the two groups within the mask. The selected areas were considered statistically significant after correction (*p* < 0.05, voxel >228) (30). All test methods were two-tailed.

## 5 Results

### 5.1 Comparison of cognitive function before and after intervention

Before treatment, the cognitive function scores of both PSCI patient groups were significantly lower compared to those of the HC group (*p* < 0.05). However, there was no significant difference between the CM group and the ER group. After 8 weeks of intervention, this difference persisted in the HC group and both PSCI groups (p < 0.05). Although both CM and ER groups exhibited significant improvement in cognitive function post-treatment (p < 0.05), the improvement in the ER group was more pronounced than that in the CM group (*p* < 0.05). Specific data are presented in Table 2.

**Table 2:** Comparison of cognitive function between three groups.

		HC group	CM group	ER group
MoCA	Pre-intervention	29.25 ± 0.75^cd^	21.75 ± 1.29^a^	21.80 ± 1.28^a^
	Post-intervention	-	23.85 ± 1.78^ab^	26.20 ± 1.64^abd^
SDMT	Pre-intervention	76.60 ± 5.75^cd^	46.90 ± 8.07^a^	46.95 ± 8.81^a^
	Post-intervention	-	57.05 ± 8.71^ab^	64.15 ± 10.11^abd^
TMT-A(s)	Pre-intervention	48.11 ± 9.04^cd^	73.25 ± 17.11^a^	73.53 ± 16.78^a^
	Post-intervention	-	72.39 ± 17.64^a^	71.76 ± 16.15^a^
TMT-B(s)	Pre-intervention	91.55 ± 20.67^cd^	149.69 ± 33.81^a^	146.85 ± 36.51^a^
	Post-intervention	-	129.71 ± 27.24^ab^	110.85 ± 17.79^abd^

^aCompared with the HC group, p < 0.05. bCompared with pre-intervention of the same group, p < 0.05. cCompared with the CM group pre-intervention, p < 0.05. dCompared with the CM group post-intervention, p < 0.05.^

### 5.2 Comparison of brain function connectivity (FC) in the ER group before and after intervention

**Figure 1:**
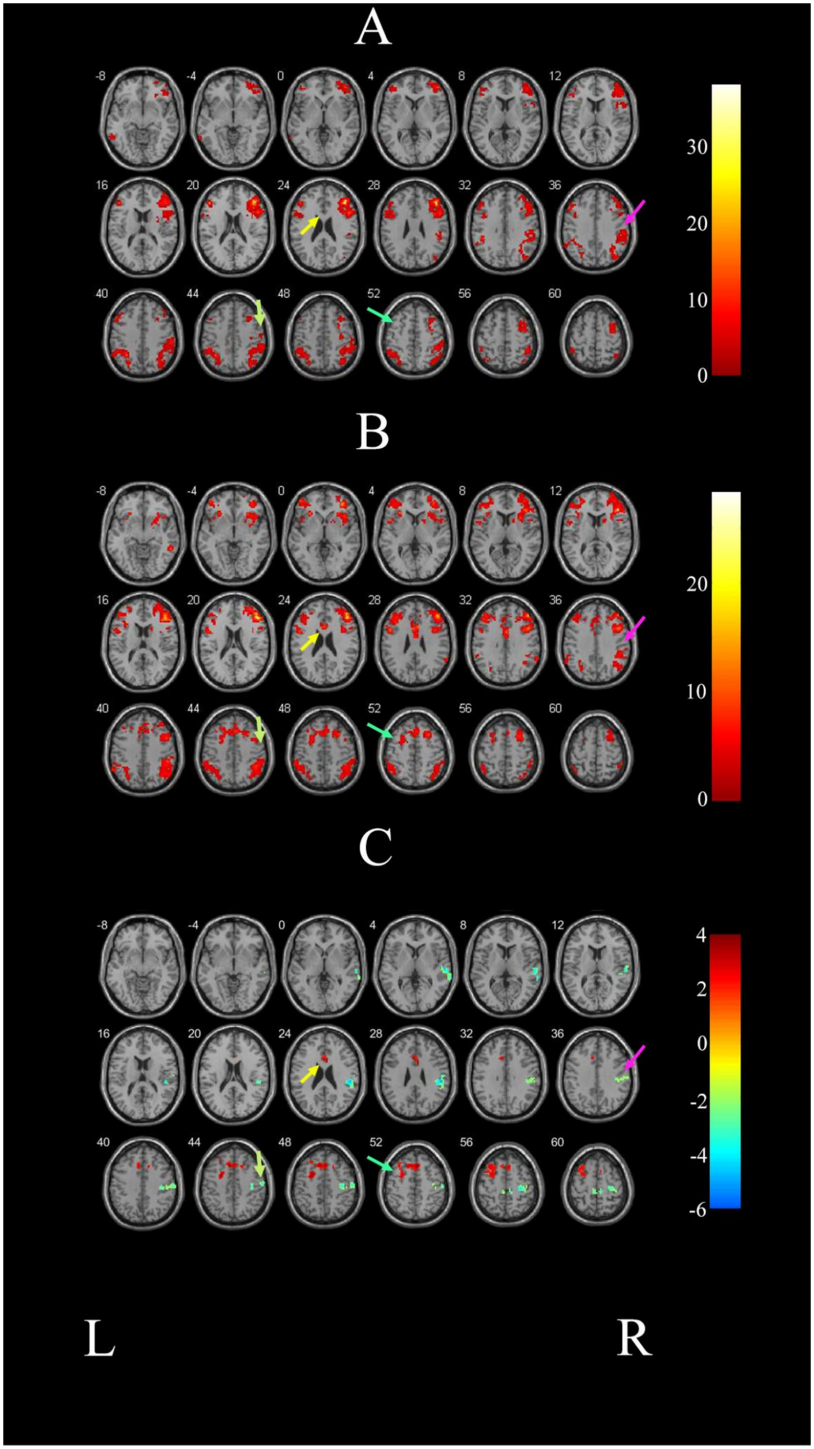
Comparison of brain FC before and after intervention in the ER group. **(A)** Illustrated the change of brain regions with FC with the right DLPFC in PSCI patients before ER intervention. **(B)** Depicts the change of brain areas exhibiting FC with the right DLPFC in the ER group after training. **(C)** Demonstrates the difference in FC before and after treatment in the ER group. The right STG is indicated by the pink arrow; the right precentral gyrus is indicated by the grassy green arrow; the left SFG is indicated by the blue-green arrow; and the left ACG is indicated by the yellow arrow. L and R represent the left and right hemispheres, respectively.

As illustrated in Figure 1A and detailed in Table 3, the FC map of the ER group before intervention revealed that the significant functional connectivity with the right DLPFC mainly encompassed the bilateral middle frontal gyrus (MFG), right middle temporal gyrus (MTG), right precentral gyrus, left inferior frontal gyrus (IFG), left parietal inferior angular gyrus, and left inferior temporal gyrus (ITG) (*p* < 0.05, voxel>228).

**Table 3:** FC of patients in the ER group before intervention.

Region	BA	MNI coordinates			T	VOX
		x	y	z		
Right MFG	45	45	36	21	37.61	1,025
Right MTG	20	57	−39	−12	5.00	384
Right precentral gyrus	6	33	−24	66	7.12	281
Left MFG	44	−51	24	36	12.24	510
Left IFG	45	−45	33	18	22.31	320
Left parietal inferior angular gyrus	40	−30	−54	39	15.91	958
Left ITG	37	−66	−54	−3	7.73	331

MFG, Middle Frontal Gyrus; MTG, Middle Temporal Gyrus; IFG, Inferior Frontal Gyrus; ITG, Inferior Temporal Gyrus; BA, Brodmann Area; T, T value; VOX, Voxel.

After the intervention, the ER group exhibited significant FC in the left anterior cingulate gyrus (ACG), right inferior temporal gyrus (ITG), bilateral middle frontal gyrus (MFG), bilateral parietal inferior angular gyrus, and the right DLPFC in the ER group (*p* < 0.05, voxel>228). These findings are illustrated in Figure 1B and detailed in Table 4.

**Table 4:** FC of patients in the ER group after intervention.

Region	BA	MNI coordinates			T	VOX
		x	y	z		
Right ITG	20	57	−45	−18	11.99	258
Left MFG	46	−36	36	30	19.53	1,050
Right MFG	45	45	33	12	28.33	1,541
Right parietal inferior angular gyrus	48	66	−48	30	14.03	999
Left parietal inferior angular gyrus	40	−57	−54	39	6.88	698
Left ACG	24	−3	12	27	14.00	173^a^

ITG, Inferior Temporal Gyrus; MFG, Middle Frontal Gyrus; ACG, Anterior Cingulate Gyrus; BA, Brodmann Area; T, T value; VOX, Voxel. ^a^The left ACG was connected to the left MFG.

Figure 1C and Table 5 demonstrate that the FC map of the ER group between the right DLPFC and deep brain regions, including the left superior frontal gyrus (SFG) and the left anterior cingulated gyrus (ACG) post-treatment, was stronger post ER compared with pre-ER (p < 0.05, voxel>228). Conversely, compared with pre-ER, the FC of post ER between the right DLPFC and the right superior temporal gyrus (STG) and the right precentral gyrus was significantly weaker (p < 0.05, voxel>228).

**Table 5:** Brain regions with differences in FC before and after treatment in the ER group.

Region	BA	MNI coordinates			T	VOX
		x	y	z		
Right STG	22	69	−39	6	−6.05	538
Right precentral gyrus	6	42	3	42	−2.34	131^a^
Left SFG	8	6	21	54	4.01	318
Left ACG	24	−3	18	27	3.09	93^b^

STG, Superior Temporal Gyrus; SFG, Superior Frontal Gyrus; ACG, Anterior Cingulate Gyrus; BA, Brodmann Area; T, T value; VOX, Voxel. ^a^The right precentral gyrus was connected to the right STG. ^b^The left SFG was connected to the left ACG.

## 6 Discussion

The present findings indicate that the implementation of ER intervention yields a notable improvement in cognitive functioning among post-stroke patients, particularly in overall cognitive function and attention executive function. This improvement is attributed to the ER intervention’s capability to enhance the FC of cognitive-related brain areas, such as the frontal cortex and anterior cingulate cortex, with the dominant hemisphere. Simultaneously, it weakens the FC with the non-dominant hemisphere, restoring the balance between bilateral hemispheres in PSCI patients. Ultimately, this fosters the establishment of a brain remodeling mechanism for cognitive function.

The behavioral findings demonstrated a significant advancement in cognitive function among post-stroke patients undergoing ER intervention, surpassing the results of conventional cognitive rehabilitation training. This improvement was particularly pronounced in attention and executive function. Concurrently, the imaging results of this study revealed alterations in brain functional network connectivity post-treatment for patients in the ER group compared to their pre-treatment conditions. Specifically, an increase in FC was observed between the right DLPFC and the left SFG as well as the left ACG, which are closely associated with attention and executive function. The rationale behind the cognitive function improvement can be attributed to the potential of ER intervention in restructuring the brain’s functional network in stroke patient (31, 32).

ER intervention, as a comprehensive training method based on an enriched environment, plays an important role in enhancing both brain plasticity and behavior (33). The research group employed a combination of multi-sensory stimulation, cognitive function training, task-oriented training, and social training to augment the rehabilitation program for ER patients. As a result, there was a significant improvement in the overall cognitive function of the patients. This improvement can be attributed to several factors. The diverse array of multi-sensory stimuli, including visual, auditory, olfactory, and tactile inputs, significantly enhances individuals’ ability to perceive and engage with their physical and social surroundings. Moreover, it also promotes neuroplasticity in the brain (33, 34). For instance, visual stimulation can facilitate the establishment of novel neural circuits for information processing and analysis, expedite the reorganization of neural function, and contribute to circuit reconstruction, thereby enhancing individuals’ adaptability to complex and dynamic environments. Furthermore, some studies have also demonstrated that visual stimulation can elicit hippocampal neurogenesis, further augmenting cognitive function (35). Cognitive function training strategically assigns appropriate cognitive tasks to patients systematically and comprehensively. Diverging from conventional cognitive rehabilitation training, ER intervention tailors cognitive rehabilitation tasks to be patient-oriented, integrating multiple cognitive functions simultaneously instead of conducting targeted independent training in isolated domains. This approach is more conducive to the comprehensive improvement of patients’ diverse cognitive functions. Concurrently, task-oriented training integrates motor and cognitive functions, activating the motor control and attention executive function networks simultaneously, thereby benefiting the enhancement of patients’ attention function, planning, and logic (36). In contrast, social training activities such as recitation, shopping, and competitive events (e.g., playing chess) contribute to the improvement of memory and other cognitive functions (37).

Studies have demonstrated that the change of cognitive function is inevitably accompanied by the remodeling of brain function (18). To explore alterations in brain regions associated with cognitive function, this study employed a relatively safe and reliable method rs-fMRI, widely acknowledged for investigating the mechanisms of brain function remodeling in stroke patients (38). In this study, the right DLPFC was chosen as the seed voxel for whole-brain FC. The selection of the DLPFC as the seed region is grounded in its significance for cognitive function, encompassing attention, working memory, executive function, and various other cognitive functions (39). Furthermore, the right DLPFC was specifically designated as the seed voxel due to the subjects’ left hemisphere stroke, which resulted in the compromised functional network of the left hemisphere. In some instances, both the structure and function of the DLPFC may have been impaired as a consequence.

The present study identified a robust connection between the right DLPFC and the frontal cortex in patients with PSCI, both before and after ER intervention. The frontal cortex, known for high-level cognitive control and sensorimotor integration, is closely related to attention and executive function. Therefore, even before ER intervention, the DLPFC exhibited FC with the frontal cortex, including the bilateral MFG and the right precentral gyrus of the non-dominant hemisphere (40–42). Radiographic results of this study revealed a significant improvement in FC between the DLPFC and the frontal cortex of the dominant hemisphere following ER intervention. We posit that this enhancement can be attributed to the ER intervention involving concentration, simulation, planning of relevant motor behaviors, among other factors, fully activating the functional activity of cognitive brain areas (24, 32).

The ACG is recognized as part of the limbic system, closely associated with attention and executive function (43, 44). Groups with a well-developed ACG are more adept at maintaining focus, filtering out external distractions, and transitioning quickly and orderly between different tasks (45, 46). Research indicates that the ACG can implement directional behavior monitoring for ongoing tasks, promptly signaling responses in the event of a conflict or error to adjust and allocate attention resources accordingly (47). Consistent with previous studies, there was no significant FC observed between the right DLPFC and ACG before ER intervention. However, following the intervention, there was a notable enhancement in the FC between the two regions. Furthermore, the patients’ test scores for attention and executive function demonstrated improvement, reinforcing the notion that ER training has the potential to optimize the FC of the attention and executive function network post-stroke.

Additionally, this study revealed that following ER intervention, there was an enhancement in the connectivity of DLPFC brain areas associated with cognitive function in the dominant hemisphere (left frontal cortex and left ACG). Simultaneously, a significant decrease was observed in the FC of cognitive-related brain areas (STG and precentral gyrus) in the non-dominant hemisphere, indicating a shift toward cognitive processing favoring the dominant hemisphere. In this study, we found that for patients with moderate PSCI, the core cognitive region DLPFC needed to enhance its FC with non-cognitive brain regions (such as the temporal cortex and precentral gyrus in the non-dominant hemisphere) to sustain cognitive activity before training. The temporal cortex is mainly related to long-term memory, while the precentral gyrus is primarily related to motor function (48, 49). The disruption of interhemispheric connectivity occurs simultaneously with stroke in the dominant hemisphere, resulting in an increased reliance on the non-dominant hemisphere for executive attention and FC (50, 51). Nevertheless, the behavioral test results suggest that the impact of this change in FC is suboptimal, indicating poor remodeling of the brain’s functional network in PSCI patients. The transformed FC is insufficient to suppress interference caused by the functional network connectivity unrelated to cognition, nor can it effectively activate the brain’s functional network involved in cognitive tasks. Following ER intervention, the cognitive function-related brain areas in the dominant hemisphere can be restored, facilitating the reestablishment of interhemispheric connection and gradual normalization of connectivity within the attention and executive brain function network. However, it was observed that the FC between DLPFC, the core region of the executive attention network, and non-dominant cognitive brain regions (such as the temporal lobe and precentral gyrus of the non-dominant hemisphere) gradually weakens. Consequently, there is a gradual shift of responsibility for cognitive functions, particularly attention and executive functioning, back to the dominant hemisphere along with notable improvements in cognitive function test scores.

The present study also has certain limitations. First of all, the absence of imaging examination for subjects in both the HC group and CM group resulted in a lack of statistical analysis and comparison concerning brain FC before and after training across these three groups of subjects. To address this limitation, the research team plans to enhance imaging examinations for subjects in each group in the subsequent experiments, aiming to investigate the similarities and differences in brain FC between PSCI patients and normal individuals before and after ER intervention. And in the follow-up study, we will continue to explore the differences between ER training and traditional training for altering functional brain connectivity, so as to provide a better basis in clinical work. Additionally, In previous studies, it has been demonstrated that hemodynamic lag interferes with FC measurements (52, 53). The disadvantage of this study is that no imaging correction was performed, thus the effect of hemodynamic lag and intrinsic cerebrovascular reactivity on functional connectivity after stroke could not be ignored, and there was some discrepancy in temporal sensitivity, which will be compensated for by combining functional near-infrared spectroscopy (fNIRS) in the follow-up study to correlate real-time functional connectivity with behavioral assessment. Furthermore, the limited conditions such as stroke location, degree of cognitive impairment, and timing of rehabilitation intervention resulted in a small sample size for each of the three experimental groups. As a result, the comprehensive exploration of the effect of ER intervention on PSCI patients was hindered. Future research endeavors should aim to expand the sample size to enhance the robustness of the study. Finally, this study did not conduct a follow-up to observe the long-term effect. In future research, the follow-up duration for patients will be extended to comprehensively assess sustained efficacy and dynamic changes in fMRI, thereby providing a more robust theoretical foundation for understanding brain remodeling mechanisms following stroke.

## 7 Conclusion

ER intervention is more effective than conventional medical rehabilitation in improving the cognitive function of PSCI patients. ER intervention has the capacity to enhance the FC between the right DLPFC and dominant cognitive brain regions, such as the left SFG and left ACG, while diminishing the FC between the DLPFC and areas of the non-dominant hemisphere, such as the STG and precentral gyrus within the right hemisphere. This reshaping of the cognitive function network contributes to a discernible improvement in cognitive function among PSCI patients.

## Data Availability

The original contributions presented in the study are included in the article/supplementary material, further inquiries can be directed to the corresponding authors.

## Footnotes

^1^Version 12; accessible at http://www.fil.ion.ucl.ac.uk/spm on May 1, 2023.

^2^Version 6.1; available at http://restfmri.net/forum/restplus on January 1, 2022.
